# Presence of a CMR-based U-shaped contraction pattern and optimal LV pacing lead position determines best response to CRT

**DOI:** 10.1186/1532-429X-16-S1-O52

**Published:** 2014-01-16

**Authors:** Gregory Hartlage, Jonathan D Suever, Stephanie Clement-Guinaudeau, Patrick T Strickland, Patrick Magrath, Michael Lloyd, John Oshinski

**Affiliations:** 1 Department of Radiology and Imaging Science, Emory University School of Medicine, Atlanta, Georgia, USA; 2 Department of Medicine, Division of Cardiology, Emory University School of Medicine, Atlanta, Georgia, USA; 3 Department of Biomedical Engineering, Georgia Institute of Technology/Emory University School of Medicine, Atlanta, Georgia, USA

## Background

Cardiac resynchronization therapy (CRT) improves outcomes in many heart failure patients, yet 1 in 3 patients do not positively respond. A "U-shaped" left ventricular (LV) activation pattern (type II), suggestive of electrical conduction block, can be characterized by cardiovascular magnetic resonance (CMR) and has been associated with improved CRT response compared to a more homogenous (type I) activation pattern. In other studies, targeting the LV pacing lead to the latest site of LV activation has been associated with improved response. We hypothesized that patients with CMR derived type II ventricular activation pattern and a concordant LV lead placement would have superior CRT response.

## Methods

**Figure 1:**
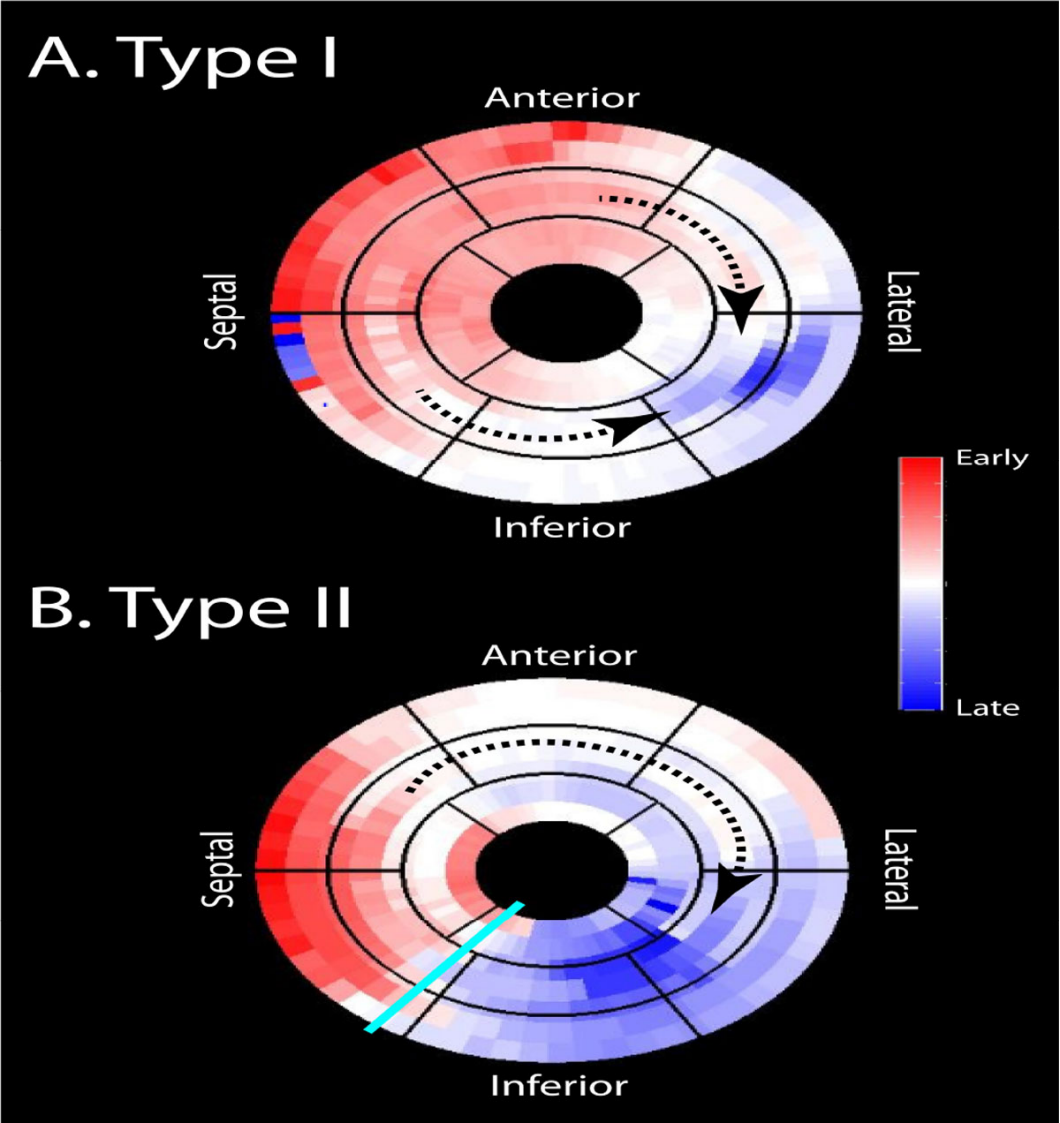
**Type I and Type II LV contraction patterns**. AHA 17-segment models demonstrating (A) Type I pattern, with dotted lines indicating homogenous wave fronts anteriorly and inferiorly towards the lateral wall and (B) Type II pattern with an inferior line of block (cyan line) and dotted line indicating only anterior wave front and late inferior wall contraction.

Patients meeting current CRT criteria were enrolled and CMR performed before the procedure on a 1.5T system. Endocardial borders were traced on short-axis cine images and radial displacement curves (RDCs) were generated and mapped to an AHA 17-segment model. LV contraction patterns were categorized as type I if the activation wave front proceeded homogenously from the septum to the free wall and type II if heterogeneous with a line of block (see Figure 1). Biplane catheter coronary venography defined coronary venous anatomy and fluoroscopically identified final LV lead position, which was mapped to the AHA 17-segment model. Final LV lead placement was considered *concordant *if within 1 segment of the latest contracting segment, and *remote *if > 1 segment from the latest contracting segment. Patients underwent echocardiography at baseline and 6 months post-implant. Positive response was reverse remodeling defined as ≥ 15% reduction in end-systolic volume (ESV) at 6 months.

## Results

27 patients (age 65 ± 12, 56% male, 81% non-ischemic) were included in the study: QRS duration was 162 ± 17 ms; EF was 28.6 ± 8.8%; typical LBBB was present in 56% of patients. On CMR imaging, 41% had type I pattern and 59% had type II pattern. Overall, the LV lead was concordant in 73% of type I patients and 63% of type II patients (p = 0.69). The overall echocardiographic response rate was 52%. The response rate for all type II pattern patients was 63%, and the response rate for all concordant lead position patients was 67%. *The response rates for type II pattern patients with concordant leads was 90%, compared to 29% for all others (p < 0.005) (see *Table 1*)*. The response rates by various EKG criteria are shown in the Table.

## Conclusions

The highest response rate to CRT among patients meeting current eligibility criteria was found in patients with "U shaped" (type II) LV contraction pattern identified by CMR and a concordant LV lead position.

## Funding

JS: National Science Foundation (NSF) fellowship. JO: National Institutes of Health (NIH) and American Heart Association (AHA) grants.

**Table 1:** Response rates by various EKG and CMR criteria.

Criteria	Marker (n)	Response Rate (↓ESV ≥ 15%)	Significance
	All patients (27)	52%	--

**EKG**	Typical LBBB (15)	60%	p = 0.45
	All others (12)	42%	
	QRS ≥ 150 ms (17)	47%	p = 0.69
	QRS < 150 ms (10)	60%	
	Typical LBBB and QRS ≥ 150 ms (9)	56%	p = 1.00
	All others (18)	50%	

**CMR**	Type I pattern (11)	36%	p = 0.25
	Type II pattern (16)	63%	
	Lead concordant (18)	67%	p < 0.05
	Lead remote (9)	22%	
	Type II pattern and lead concordant (10)	90%	p < 0.005
	All others (17)	29%	

EKG = electrocardiogram; CMR = cardiovascular magnetic resonance; LBBB = left bundle branch block; QRS = QRS complex duration

